# Sphingomyelin 16:0 is a therapeutic target for neuronal death in acid sphingomyelinase deficiency

**DOI:** 10.1038/s41419-023-05784-2

**Published:** 2023-04-06

**Authors:** Ángel Gaudioso, Xuntian Jiang, Josefina Casas, Edward H. Schuchman, María Dolores Ledesma

**Affiliations:** 1 Centro Biologia Molecular Severo Ochoa (CSIC-UAM), 28049 Madrid, Spain; 2 Washington University in St. Louis School of Medicine, St. Louis, MO USA; 3 RUBAM, IQAC-CSIC & CIBEREHD, 08034 Barcelona, Spain; 4 Department of Genetics & Genomic Sciences, Icahn School of Medicine at Mount Sinai, New York, NY USA

**Keywords:** Diseases of the nervous system, Neurological disorders

## Abstract

Acid sphingomyelinase deficiency (ASMD) is a lysosomal storage disorder caused by mutations in the *SMPD1* gene encoding for the acid sphingomyelinase (ASM). While intravenous infusion of recombinant ASM is an effective treatment for the peripheral disease, the neurological complications of ASMD remain unaddressed. It has been shown that aberrantly high level of total brain sphingomyelin (SM) is a key pathological event leading to neurodegeneration. Using mice lacking ASM (ASMko), which mimic the disease, we here demonstrate that among the SM species, SM16:0 shows the highest accumulation and toxicity in ASMko neurons. By targeting lysosomes, SM16:0 causes permeabilization and exocytosis of these organelles and induces oxidative stress and cell death. We also show that genetic silencing of Ceramide Synthase 5, which is involved in SM16:0 synthesis and overexpressed in the ASMko brain, prevents disease phenotypes in ASMko cultured neurons and mice. The levels of SM16:0 in plasma also show a strong correlation with those in brain that is higher than in liver, even at early stages of the disease. These results identify SM16:0 both as a novel therapeutic target and potential biomarker of brain pathology in ASMD.

## Introduction

Sphingomyelin (SM) is one of the most abundant sphingolipids in cellular membranes. It consists of a polar head group, phosphocholine, and a hydrophobic backbone, ceramide [1]. Its chemical properties, together with its significant interactions with cholesterol, contribute to the formation of membrane microdomains that serve as signalling platforms [2]. Moreover, SM metabolism produces important second messengers (e.g., ceramides) for signal transduction [3]. Synthesis and degradation of SM is tightly controlled by a complex array of enzymes, which in mammalian cells include different isoforms of ceramide synthases (CerS), sphingomyelin synthases (SMS) and sphingomyelinases (SMases). These enzymes show differences in their levels of expression depending on the tissue, cell type and subcellular compartment [4–7]. Adding further complexity, numerous SM species exist that differ in the length and degree of unsaturation of their fatty acids, and these different species have distinct subcellular localization, metabolism and properties [8–10]_._

SM is particularly enriched at the plasma and synaptic membranes of neurons. Besides its involvement in synapses by modulating neurotransmitter receptor physiology [11] and dendritic spine dynamics [12], SM contributes to other important physiological processes in neurons such as the establishment of axonal polarity [13, 14], autophagy [15], and calcium homeostasis [16]. In agreement with these important roles, alterations in neuronal SM levels lead to severe neurological diseases. Among them is the infantile neurovisceral form of the acid sphingomyelinase deficiency (ASMD), also called Niemann Pick type A. Loss of function mutations in the sphingomyelin phosphodiesterase 1 (*SMPD1*) gene encoding acid spingomyelinase (ASM) cause severe neurological disease, neurodegeneration and death in the first years of life [17]. Intermediate and non-neurologic forms of the disease also have been described (type A/B and type B, respectively) [17].

Aberrantly high SM levels are a hallmark of all ASMD cells. Studies performed in mice lacking the ASM (ASMko), which faithfully mimic neurovisceral ASMD [18], have shown that increased SM levels are directly responsible for many pathological phenotypes in neurons [19]. Intravenous infusion of recombinant ASM has proven successful to treat peripheral ASMD disease in mouse models [20] and patients [21, 22]. However, the recombinant ASM does not cross the blood brain barrier (BBB) and therefore leaves the neurological phenotype unaddressed. Most, if not all, studies in ASMD have referred to the deleterious effects of the increase in the levels of total SM in the brain. However, analysis of potential changes in the different SM species and metabolic enzymes, and their contribution to toxicity in ASMD is still lacking. This information may reveal new strategies aimed at reducing specifically certain species, and provide new insights regarding the underlying CNS pathology in this disease. In addition, altered levels of these species in cerebrospinal fluid (CSF) and/or plasma could become indicators for brain disease and therapy response in ASMD, which are currently lacking.

## Materials and methods

### Antibodies

Antibodies against the following proteins were used in Western blot and immunohistochemical and immunocytochemical analysis: β-Actin (Merck; #A5441); Calbindin (Swant; #300); Cathepsin B (R&D Systems; #AF965); CerS5 (Invitrogen; #PA5-20569); CerS6 (Santa Cruz; #sc100554); EEA1 (R&D Systems; #MAB8047); F4/80 (Abcam; #ab6640); GAPDH (Abcam; #ab8245); GFAP (Merck; # MAB3402); GM130 (BD; #610823); Iba1 (Wako; #019-19741); Lamp1 (DSHB; #1D4B); LSDP5 (Abcam; #ab222811); MAP2 (Biolegend; #822501); PSD95 (Neuromab; #75-028); TOM20 (Santa Cruz; #sc-11415).

### Mice

Breeding colonies were established from ASM heterozygous C57BL/6 mice [18], kindly donated by Prof. EH Schuchman (Mount Sinai School of Medicine, New York, NY, USA). Male and female ASMko and wild-type (wt) littermates were analyzed at 4.5 months of age. No gender-dependent differences were observed in any of the results. Procedures followed European Union and the ARRIVE guidelines and were approved by the CBMSO Animal Welfare Committee.

### Neuronal cultures

Primary cultures of cortical neurons were prepared from day 17 wt and ASMko embryos as described [23]. Neurons were kept under 5% CO_2_ at 37 °C in Neurobasal Medium (Gibco) plus B27 Supplement (Gibco) and GlutaMAX (Gibco) until 7 days in vitro (DIV). The medium was then replaced with Neurobasal Medium plus B27 without GlutaMAX.

### In vitro treatments

Cultured neurons were treated at DIV12 for 48 h with a stock solution of SM dissolved in 100% ethanol reaching a final concentration of 40 µM. Control neurons were treated with the same volume of vehicle (100% ethanol). Final volume of ethanol was never higher than 1% of the total cell culture media volume to avoid toxicity. Sphingomyelins used in this study were: SM from bovine brain (Santa Cruz; #sc-201381); SM16:0 (Merck; #860584 P); SM18:0 (Merck; #860586 P) and SM24:1 (Merck; #860593 P). Lyso Sphingomyelin (Merck; #860600 P; 40 µM); Desipramine (Merck; # D3900; 10 µM) and GW4869 (Cayman Chemicals; #13127; 15 µM) were added, from stocks dissolved in 100% ethanol, to cultured neurons 48 h before processing.

### Quantification of SM species in the brain and liver of ASMko mice

Mouse brain and liver samples were homogenized in water (1 g wet tissue/4 mL). SM was extracted from 50 μL of homogenate or 50 μL of plasma in the presence of SM (17:0) as internal standard using Bligh-Dyer lipid extraction [24]. Sample analysis was performed in a Shimadzu 20AD HPLC system coupled to a 6500QTRAP + mass spectrometer (AB Sciex, Framingham, MA) operated in positive multiple reaction monitoring mode. Data processing was conducted with Analyst 1.6.3 (AB Sciex). Data were reported as the peak area ratios of the analytes in sample to the internal standard.

### Quantification of SM species in cultured neurons

Sample analysis was performed in an Acquity ultraperformance liquid chromatography (UPLC) system (Waters, USA) connected to a time-of-flight (TOF; LCT Premier XE) detector controlled with Waters/Micromass MassLynx software.

### Quantification of total SM by enzymatic assay

SM levels were measured according to protocols modified from [25]. Lipid extracts were dried in the presence of Thesit, and SM was converted into peroxide by incubation with sphingomyelinase (Smase C from Merck; #S9396 or ASM from Sino Biological; #50749-M08B), alkaline phosphatase, and choline oxidase. Peroxide was measured fluorimetrically in the presence of peroxidase and homovanillic acid [26].

### Immunohistochemical and immunocytochemical analysis

Cultured neurons at 14 DIV were fixed in 4% paraformaldehyde (PFA) 0.12 M sucrose and incubated overnight with primary antibodies and subsequently for 1 h with Alexa 488- or Alexa 555-conjugated secondary antibodies. Images were taken using a confocal microscope LSM710 (Zeiss). The Mander’s coefficient for pSM 16:0 co-localization with PSD95; TOM20; EEA1; LSDP5; LAMP1; GM130 was determined by using the JACoP plugin. The Mander’s coefficient for Cathepsin B co-localization with LAMP1 was determined by using the JACoP plugin.

Mouse brains were dissected, fixed in 4% PFA 0.12 M sucrose, and cryoprotected for 24 h in 30% sucrose phosphate buffer saline. The tissue was then frozen in Tissue-Tek optimal cutting temperature compound (Sakura Finetek, Torrance, CA, USA), and 40-µm sagittal sections were obtained with a cryostat (CM 1950 Ag Protect freezing: Leica, Solms, Germany). The sections were incubated overnight at 4 °C with the primary antibodies and then with the corresponding Alexa-conjugated secondary antibodies. Finally, the sections were incubated for 10 min with DAPI (Merck), washed, and mounted with ProLong Gold Antifade (Thermo Fisher). Images were obtained on a confocal LSM710 microscope (Zeiss) and quantified using the Fiji software.

### Reactive oxygen species determination

To assess reactive oxygen species (ROS), cultured neurons were incubated for 20 min with 10 μΜ dihydrorhodamine 123 (DHR) (Molecular Probes, Carlsbad, CA, USA). The fluorescence product of DHR, rhodamine-123, was detected at excitation and emission wavelengths of 500 and 536 nm, respectively. Images of cultured neurons were captured on a confocal microscope (LSM710; Zeiss).

### Cell viability assay

Cell viability was evaluated using the MTT (methylthiazolyldiphenyltetrazolium bromide, Sigma) assay. Briefly, 3 × 10^4^ neurons were plated in 96-well plates coated with poly-L-lysine (0.1 mg/ml). At DIV14 1 mM MTT was added to the wells and incubated for 4 h. Then, the media was discarded, and 500 µl DMSO was added to the wells. The absorbance was measured at 570 nm using a microplate reader (FLUOstar Optima, BMG Labtech). Cells treated with DMSO were used as a control.

### Apoptosis assay

Percentage of apoptotic cells was determined using DeadEnd™ Fluorometric TUNEL (TdT-mediated dUTP Nick-End Labeling) System (Promega; # G3250) following the manufacturer instructions.

### Propargyl SM

Cultured neurons were treated with 40 µM propargyl SM (pSM 16:0) (Merck; # 860711 P) for 48 h at DIV12. After running the immunocytochemical protocol, explained above, click reaction was performed for the attachment of the fluorescent reporter (Sulfo-Cy3-Azide; Jena Biosciences; #CLK-AZ119-1) to pSM 16:0 as indicated for the fluorescent detection of other alkyne lipids [27].

### Quantitative RT–PCR

Total RNA from wt and ASMko cerebellum and cortex was extracted with TRIzol Reagent (Ambion/RNA Life Technologies Co.) following the manufacturer instructions. RNA was quantified by absorbance at 260 nm using a NanoDrop ND-100 (Themo Fisher Scientific Inc.). Retrotranscription to first-strand cDNA was performed using RevertAid H Minus First-Strand cDNA Synthesis Kit (Thermo Fisher Scientific Inc.). Briefly, 10 ng of synthesized cDNA were used to perform fast qPCR using GoTaq qPCR Master Mix (Promega Co., Madison, WI, USA) in ABI PRISM 7900HT SDS (Applied Biosystems; Life Technologies Co.) following manufacturer instructions.

The primers corresponding to the genes encoding the different SM metabolic enzymes (listed in Table 1) were purchased from Life Technologies and used at 0.5 µM final concentration. Three housekeeping genes (Gapdh, Gusb and Pgk1) were used as endogenous controls.

**Table 1:** Primers used in the qPCR experiments.

Gene	Encoded protein	Forward primer	Reverse primer
CerS1	CerS1	5′-GCATCAGTGCCCTGTACTGT-3′	5′-GACCAACTTCTACCCTGGGC-3′
CerS2	CerS2	5′-AATAACAATCATCCTAAGAAT-3′	5′-TAGACTCCTCATATCCTT-3′
CerS3	CerS3	5′-CAACTTTCGAGGGTGGGTCT-3′	5′-TTCTTCTTCCCAAAGCGCCA-3′
CerS4	CerS4	5′-TTGCCTTAGTCTTCTTCT-3′	5′-GATGGAGTCATACACAGA-3′
CerS5	CerS5	5′-TCTACACTCCTGTGACTA-3′	5′-AGCCAATAACCTTACTGAA-3′
CerS6	CerS6	5′-TACTGCTCTGACGACTTG-3′	5′-GCTGTTGCTGCTATTCTC-3′
Sgms1	SMS1	5′-CGTTGGAATCTTCTGTAT -3′	5′-CTTGTGGTGATGTAGTAG-3′
Sgms2	SMS2	5′-AAAGGCCACCTGTCTGTCC-3′	5′-GGCCTGACCAATGCTCTCTT-3′
Smpd2	nSMase-1	5′-TGGGACATCCCCTACCTGAG-3′	5′ TAGGTGAGCGATAGCCTTTGC-3′
Smpd3	nSMase-2	5′-ACACGACCCCCTTTCCTAATA-3′	5′-GGCGCTTCTCATAGGTGGTG-3′
Smpd4	nSMase-3	5′-CACACTAGCCTCTTGAAGCGA-3′	5′-TGTAGAACCTCCAACTTGGCAT-3′

*SMS* sphingomyelin synthase, *nSMase* neutral sphingomyelinase.

### Adenoviral infection in vitro

Cultured neurons from wt mice were treated with adenovirus containing shRNA-scramble or shRNA-CerS5 at a multiplicity of infection (MOI) = 20. Viral infection was done at 6DIV and for 24 h.

### AAV9 infection in vivo

AAV9 shRNA-scramble or shRNA-CerS5 (2 µl; 5 × 10^12^ VG/ml) virus diluted in artificial cerebrospinal fluid (aCSF) was injected at 0.2 µl/min by a glass micropipette into the deep cerebellar nucleus of both hemispheres [anterior-posterior (AP): − 5.75 mm; medial-lateral (ML): ± 1.8 mm; dorso-ventral (DV): − 2.6 mm] in mice anesthesized by intraperitoneal injection of thiobarbital (5% dissolved in distilled water) and intranasal infusion of isofluorane. Virus injection was performed at 8 weeks of age. After surgery, mice were placed in a recovery chamber and were monitored until fully recovered. Then, mice were transferred to their home cages and were monitored daily by trained personnel during the first week after injection and then weekly for the following 7 weeks.

### Statistical and correlation analysis

Data from at least three different experimental groups were quantified and presented as the mean ± SEM. Sample size was chosen based on previously published experiments of the same kind. No samples were excluded from the analysis and no randomization was used. Investigators were not blinded to the mouse group allocation. Normality of the data was tested using the Shapiro–Wilk test. For two-group comparisons, the Mann–Whitney U-test for non-parametric data or a two-sample Student’s *t* test for data with parametric distribution was used. For multiple comparisons, data with a normal distribution were analyzed by one-way ANOVA followed by Tukey post hoc test. The statistical significance of non-parametric data was determined by the Kruskal–Wallis test to analyze all experimental groups. Correlation analyses were performed computing Pearson correlation coefficients with a confidence interval of 95%. Linear regression test with 95% confidence interval was used to analyze slope differences. *p* values (*p*) < 0.05 were considered significant. In the figures, asterisks indicate the *p* values: *p < 0.05; **p < 0.01; ***p < 0.001; ****p < 0.0001. GraphPad Prism 6.0 software (GraphPad Software, La Jolla, CA, USA) was used for all statistical analysis.

The statistical analysis of liver and brain SM species was performed using MetaboAnalyst 5.0 software [28]. The lipidomic data were normalized (mean-centered and divided by the standard deviation of each variable) and represented using a heatmap or a principal component analysis (PCA) after autoscale of each SM species.

## Results

### SM16:0 is the SM species with the highest relative increase in ASMko compared to wt mouse brains and neurons

**Fig. 1:**
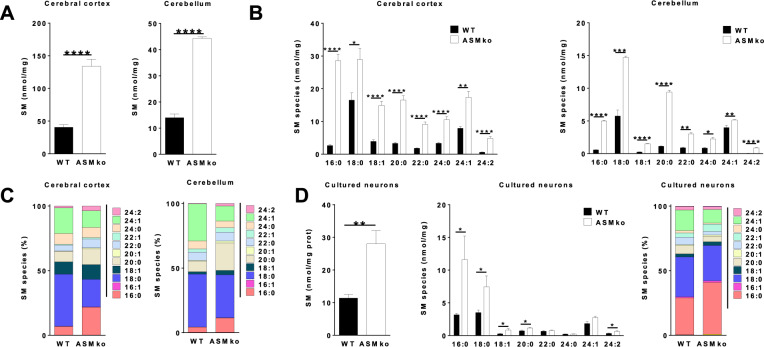
Variable relative increase of SM species in the ASMko brain and cultured neurons. **A** Graphs show mean ± SEM of total SM levels expressed as nmol/mg protein in extracts from the cerebral cortex and cerebellum of wt and ASMko mice at 4.5 months of age (*n* = 5; ****p < 0.0001). **B** Graphs show mean ± SEM levels of the indicated SM species expressed as nmol/mg protein in extracts from the cerebral cortex and cerebellum of wt and ASMko mice at 4.5 months of age (*n* = 5; *p < 0.05; **p < 0.01; ***p < 0.001; ****p < 0.0001). **C** Bars show mean percentages of the indicated SM species with respect to total SM in extracts from the cerebral cortex and cerebellum of wt and ASMko mice at 4.5 months of age (*n* = 5). **D** Graphs show mean ± SEM levels of total SM or SM species expressed as nmol/mg protein (left and middle panels) or mean percentages (right panel) of SM species with respect to total SM in extracts from cultured cortical neurons from wt and ASMko mice (*n* = 3 independent cultures; **p < 0.01).

While high levels of total SM have been reported in the brain of ASMko mice compared to wt [14, 18] little is known about the changes in specific SM species. To address this issue we analyzed the levels of total SM and of different SM species by Liquid Chromatography/ Mass Spectrometry (LC/MS) in the cerebellum and cortex of wt and ASMko mice at 4.5 months of age. At this age, neurological symptoms of the disease are evident including motor and cognitive impairment [12, 18]. The results confirmed a similar increment of total SM levels in the cortex and cerebellum of the ASMko compared to wt mice (3.3-fold and 3.2-fold, respectively) (Fig. 1A). However, while the levels of all SM species were increased, the extent of this increase was variable (Fig. 1B, C). Intriguingly, the SM species that increased less were the most abundant in wt conditions: SM18:0 (1.7-fold increase in the ASMko cortex and 2.6-fold in cerebellum) and SM24:1 (2.2-fold cortex; 1.3-fold cerebellum). In contrast, the amount of SM16:0, which exists in minor amounts in the wt situation, showed a dramatic increase (10.8-fold cortex; 8.6-fold cerebellum) (Fig. 1B). Analyses of the data in terms of relative amounts of these SM species indicated that SM18:0 and SM24:1 accounted for 60% and 70% of total SM in the wt mouse cortex and cerebellum, respectively. These percentages decreased to 33% and 44% of total SM in the cortex and cerebellum of the ASMko mouse brain (Fig. 1C). In contrast, the relative amount of SM16:0 with respect to total SM increased from less than 6% and 4% in wt cortex and cerebellum to 20% and 11%, respectively, in the ASMko mice (Fig. 1C). To confirm whether the changes observed in brain extracts also occur in neurons, we performed LC/MS analysis in primary cultures of cortical neurons derived from wt and ASMko mice (Fig. 1D). Total SM levels showed a 2.5-fold increase in the ASMko neurons compared to wt. The increment in SM 16:0 was higher (3.6-fold) than in SM18:0 (2.1-fold) and in SM 24:1 (1.5-fold) (Fig. 1D). Analysis of the relative amounts of SM species showed similar trends than in the brain. Thus, while SM18:0 and SM24:1 reduced their prevalence (by 3% and 5% respectively) the relative abundance of SM16:0 increased (by 12%) in the ASMko neurons compared to wt (Fig. 1D).

### SM16:0 is the SM species that accumulates the most and shows highest toxicity in cultured neurons

**Fig. 2:**
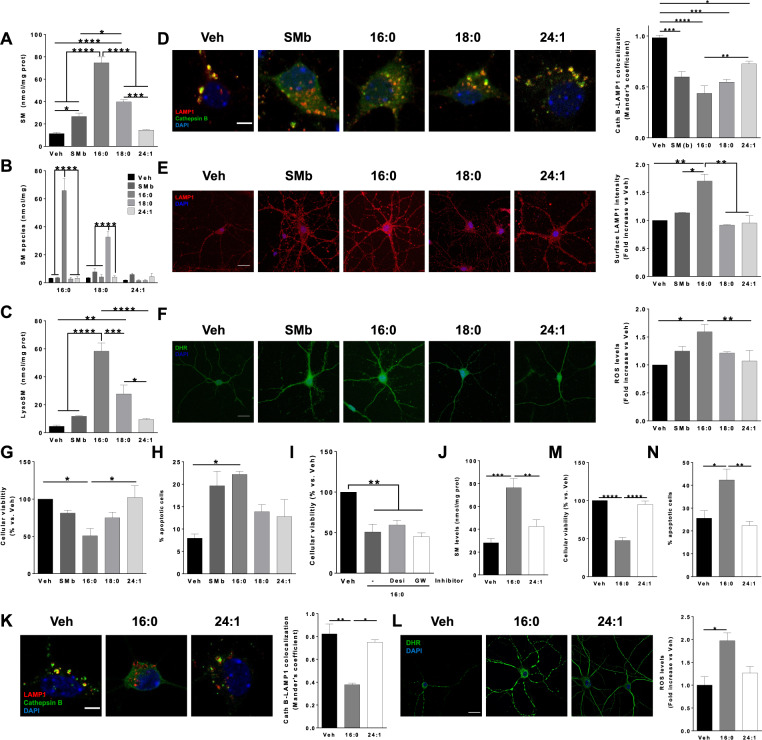
Different impact of SM species when added to cultured wt and ASMko neurons. **A** Graphs show mean ± SEM of total SM levels expressed as nmol/mg protein in cultured wt neurons incubated with vehicle or with the indicated SM species (*n* = 3 independent cultures; **p < 0.01; ***p < 0.001; ****p < 0.0001). **B** Graphs show mean ± SEM levels of the SM species indicated in the *X*-axis expressed as nmol/mg protein in extracts from wt cultured neurons treated with vehicle or with the SM species indicated in the legend to the right (*n* = 4; ****p < 0.0001). **C** Graphs show mean ± SEM levels of Lyso SM expressed as nmol/mg protein in extracts from wt cultured neurons treated with vehicle or with the indicated SM species (*n* = 4; *p < 0.05; **p < 0.01; ***p < 0.001; ****p < 0.0001). **D** Immunocytochemical analysis against the lysosomal resident protein Cathepsin B and the lysosomal marker LAMP1 in cultured wt neurons incubated with vehicle or with the indicated SM species. Graph shows mean ± SEM Mander’s colocalization coefficient between Cathepsin B and Lamp1 (*n* = 3 independent cultures; *p < 0.05; **p < 0.01; ***p < 0.001; ****p < 0.0001). DAPI staining shows cell nuclei. Bar = 5 µm. **E** Immunocytochemical analysis against the lysosomal marker LAMP1 in non-permeabilized cultured wt neurons incubated with vehicle or with the indicated SM species. Graph shows mean ± SEM LAMP1 surface staining expressed as fold-increase with respect to vehicle treated cultures (*n* = 3 independent cultures; *p < 0.05; **p < 0.01). Bar = 20 µm. **F** DHR staining in cultured wt neurons incubated with vehicle or with the indicated SM species. Graph shows mean ± SEM DHR intensity, proportional to ROS levels, expressed as fold-increase with respect to vehicle treated cultures (*n* = 4 independent cultures; *p < 0.05; **p < 0.01). DAPI staining shows cell nuclei. Bar = 20 µm. **G** Graph shows mean ± SEM cellular viability measured by MTT in cultured wt neurons incubated with vehicle or with the indicated SM species and expressed as percentage of living cells with respect to vehicle treated cultures (*n* = 3 independent cultures; *p < 0.05). **H** Graph shows mean ± SEM percentage of apoptotic cells measured by TUNEL assay in cultured wt neurons incubated with vehicle or with the indicated SM species (*n* = 3 independent cultures; *p < 0.05). **I** Graph shows mean ± SEM cellular viability measured by MTT in cultured wt neurons incubated with vehicle or with the indicated SM species in the presence of Desipramine (which inhibits the ASmase) or GW4569 (which inhibits the NSmase). Data are expressed as percentage of living cells with respect to vehicle treated cultures (*n* = 3 independent cultures; **p < 0.01). **J** Graphs show mean ± SEM of total SM levels expressed as nmol/mg protein in cultured ASMko neurons incubated with vehicle or with the indicated SM species (*n* = 3 independent cultures; **p < 0.01; ***p < 0.001). **K** Immunocytochemical analysis against the lysosomal resident protein Cathepsin B and the lysosomal marker LAMP1 in cultured ASMko neurons incubated with vehicle or with the indicated SM species. Graph shows mean ± SEM Mander’s colocalization coefficient between Cathepsin B and Lamp1 (*n* = 3 independent cultures; *p < 0.05; **p < 0.01). DAPI staining shows cell nuclei. Bar = 5 µm. **L** DHR staining in cultured ASMko neurons incubated with vehicle or with the indicated SM species. Graph shows mean ± SEM DHR intensity, proportional to ROS levels, expressed as fold-increase with respect to vehicle treated cultures (*n* = 3 independent cultures; *p < 0.05). B DAPI staining shows cell nuclei. ar = 20 µm. **M** Graph shows mean ± SEM cellular viability measured by MTT in cultured ASMko neurons incubated with vehicle or with the indicated SM species and expressed as percentage of living cells with respect to vehicle treated cultures (*n* = 4 independent cultures; ****p < 0.0001). **N** Graph shows mean ± SEM percentage of apoptotic cells measured by TUNEL assay in cultured ASMko neurons incubated with vehicle or with the indicated SM species (*n* = 3 independent cultures; *p < 0.05; **p < 0.01).

The different increment of individual SM species in ASMko mice and cells prompted us to address the impact of their specific accumulation in neurons. SMs dissolved in organic solvents can be incorporated into cells in the absence of specific carriers or detergents [12, 16]. We therefore cultured cortical neurons from wt mice for 48 h with 40 μM of either total SM from bovine brain (SMb, containing similar SM species proportions than total SM from mouse brain); or individual SM16:0; SM18:0 or SM24:1, each dissolved in 100% ethanol that was used as vehicle. LC/MS analyses revealed that while addition of SMb, SM16:0 and SM18:0 resulted in a significant increase of total SM levels (2.3-fold; 6.6-fold and 3.5-fold, respectively) in the wt cells, the increment contribution of SM24:1 to the total SM content was not significant (Fig. 2A). The increase in total SM levels was due to the accumulation of the specific SM species added in each case (Fig. 2B). Addition of SM16:0 and SM18:0 also resulted in increased amounts of lysoSM (12.7-fold and 6.0-fold, respectively (Fig. 2C), most likely as the result of the higher accumulation of these species. Accumulation of lipids in lysosomes is a pathological hallmark in ASMD cells that classifies this disease as a lysosomal storage disorder [17]. To investigate the effect of the different SM species in these organelles, we measured lysosomal-related parameters. Lysosomal permeabilization was analyzed by the co-localization of the lysosomal resident protein cathepsin B with the lysosomal associated membrane protein LAMP1. SM 16:0 promoted the highest reduction (2.2-fold) in cathepsin B-LAMP1 colocalization coefficient compared to SM18:0 (1.8-fold reduction) or SM24:1 (1.3-fold reduction), together with diffuse cytosolic Cathepsin B labelling (Fig. 2D). SM16:0 was also the only SM species that induced lysosomal exocytosis as indicated by the increased surface staining of LAMP1 in non-permeabilized neurons (Fig. 2E). Since high levels of SM also can induce oxidative stress, we measured reactive oxygen species (ROS) by dihydrorhodamine 123 (DHR) staining [16] in wt neurons incubated with the different SM species. SM16:0 induced the highest elevation in ROS levels (1.6-fold) compared to the other SM species (Fig. 2F). To determine whether different SM species could have distinct toxicity effects on neurons, we analyzed cell viability by the MTT technique. While addition of SMb and SM18:0 reduced neuronal survival by 19% and 25%, respectively, SM16:0 had a more severe impact causing the death of 50% of the seeded cells (Fig. 2G). Cell death was apoptotic as determined by the TUNEL assay (Fig. 2H). In contrast, SM24:1 had no deleterious effects on neuronal survival (Fig. 2G, H). To confirm that the cell death observed upon SM16:0 addition is due to this SM species, and not to its conversion into ceramide, we added SM16:0 in the presence of inhibitors for sphingomyelinases. We used desipramine, which interferes with the binding of ASM to lipid bilayers diminishing the contact with the substrate and therefore the activity of the enzyme [29], and the non-competitive inhibitor of the Neutral sphingomyelinase GW4869. None of the inhibitors prevented neuronal death (Fig. 2I). We next questioned whether SM 16:0 addition had similar effects on ASMko neurons. As in wt neurons, incubation of ASMko cultured cortical neurons for 48 h with 40 μM SM16:0 resulted in 2.7-fold increase in total SM levels (Fig. 2J), 1.9-fold reduction in the co-localization of Cathepsin B with LAMP1 (Fig. 2K), 2.0-fold increase in ROS levels (Fig. 2L), 1.9-fold reduction in cell survival (Fig. 2M) and 1.7-fold increase in apoptotic cells (Fig. 2N). Also as in wt neurons, addition of SM24:1 did not have effect on these parameters in the ASMko neurons (Fig. 2J–N). To determine whether ASMko neurons were more vulnerable than wt to SM 16:0 we performed dosage experiments incubating wt and ASMko neurons with 10 μM and 20 μM SM16:0. In agreement with higher susceptibility of the ASMko neurons, these cells showed higher accumulation of total SM than wt neurons when incubated with SM16:0 (1.1 and 1.4-fold SM increase in wt neurons treated with 10 μM and 20 μM SM16:0, respectively, compared to vehicle treated; 1.4 and 2.1-fold SM increase in ASMko neurons treated with 10 μM and 20 μM SM16:0, respectively, compared to vehicle treated) (Supplementary Fig. 2A). They also showed higher ROS levels (1.0 and 1.4-fold ROS increase in wt neurons treated with 10 μM and 20 μM SM16:0, respectively, compared to vehicle treated; 1.9 and 2.0-fold ROS increase in ASMko neurons treated with 10 μM and 20 μM SM16:0, respectively, compared to vehicle treated (Supplementary Fig. 2B). The TUNEL assay indicated that the percentage of apoptotic cells in vehicle treated cultures was higher in the ASMko neurons (28%) than in the wt neurons (8%). Addition of SM16:0 increased the percentage of apoptotic cells to 16% at 10 μM and 18% at 20 μM in wt neurons. In the ASMko neurons SM16:0 addition caused greater effect increasing the percentage of apoptotic cells to 43% at 10 μM and 45% at 20 μM (Supplementary Fig. 2C).

### SM16:0 accumulates in the endolysosomal compartment of cultured neurons

**Fig. 3:**
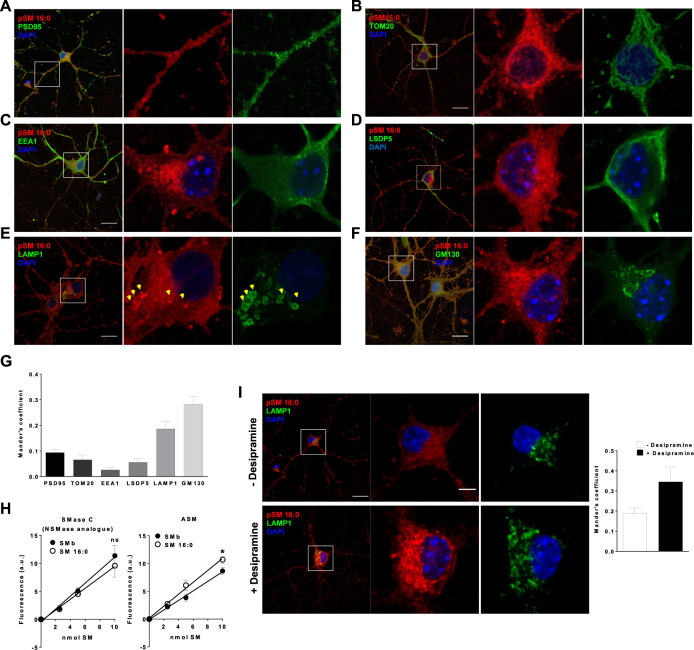
SM16:0 targets lysosomes in cultured neurons. **A** Merged low magnification image (left) and single-channel magnified insets (right) of cultured wt neurons incubated with the fluorescent analogue pSM16:0 co-stained by immunocytochemistry against the postsynaptic density marker PSD95. DAPI staining shows cell nuclei. Bar = 20 µm. Bar (high magnification) = 5 µm. **B** Merged low magnification image (left) and single-channel magnified insets (right) of cultured wt neurons incubated with the fluorescent analogue pSM16:0 co-stained by immunocytochemistry against the mitochondrial marker TOM20. DAPI staining shows cell nuclei. Bar = 20 µm. Bar (high magnification) = 5 µm. **C** Merged low magnification image (left) and single-channel magnified insets (right) of cultured wt neurons incubated with the fluorescent analogue pSM16:0 co-stained by immunocytochemistry against the early endosomal marker EEA1. DAPI staining shows cell nuclei. Bar = 20 µm. Bar (high magnification) = 5 µm. **D** Merged low magnification image (left) and single-channel magnified insets (right) of cultured wt neurons incubated with the fluorescent analogue pSM16:0 co-stained by immunocytochemistry against the lipid droplet marker Perilipin 5 (LSDP5). DAPI staining shows cell nuclei. Bar = 20 µm. Bar (high magnification) = 5 µm. **E** Merged low magnification image (left) and single-channel magnified insets (right) of cultured wt neurons incubated with the fluorescent analogue pSM16:0 co-stained by immunocytochemistry against the late endosome-lysosomal marker LAMP1. DAPI staining shows cell nuclei. Bar = 20 µm. Bar (high magnification) = 5 µm. **F** Merged low magnification image (left) and single-channel magnified insets (right) of cultured wt neurons incubated with the fluorescent analogue pSM16:0 co-stained by immunocytochemistry against the Golgi marker GM130. DAPI staining shows cell nuclei. Bar = 20 µm. Bar (high magnification) = 5 µm. **G** Graph shows mean ± SEM Mander´s coefficient of colocalization between pSM16:0 and the indicated subcellular markers PSD95, TOM20, EEA1, LSDP5, LAMP1 or GM130 (*n* = 3 independent cultures). **H** Graph shows mean ± SEM levels of fluorescence in arbitrary units with respect to nmol of SMb or SM16:0 metabolized by Smase C (NSmase analogue) or ASM activity (*n* = 3 independent experiments; *p < 0.05). **I** Images show pSM16:0 colocalization with the lysosomal marker LAMP1 in wt neurons treated or not with the ASM inhibitor desipramine. DAPI staining shows cell nuclei. Graph shows mean ± SEM Mander´s coefficient of colocalization between pSM16:0 and LAMP1 (*n* = 3 independent cultures).

The remarkable effects of SM16:0 on lysosomes compared to other SM species led us hypothesize that this lipid targets these organelles. To explore this possibility, we took advantage of the existence of the analogue propargyl SM16:0 (pSM16:0), which is linked to an alkyne group and can be traced by click chemistry and fluorescence microscopy [30]. We added pSM16:0 for 48 h to cultured wt neurons and performed double immunocytochemical analysis with markers of different cellular compartments. Analysis of the Mander’s coefficient (Fig. 3G) indicated a low degree of co-localization of pSM16:0 with the synaptic marker PSD95 (Fig. 3A), the mitochondrial marker TOM20 (Fig. 3B), the early endosome marker EEA1 (Fig. 3C) or the lipid droplet marker perilipin 5 (Fig. 3D). In contrast, pSM16:0 showed a high colocalization with the endolysosomal marker LAMP1 (Fig. 3E) and the Golgi apparatus marker GM130 (Fig. 3F). These results supported that late endosome and lysosomes are a target organelle for SM16:0 in neurons. Consistent with the preferential localization of SM16:0 in acidic compartments like lysosomes, we observed a higher efficacy of the ASM, compared to NSM, to degrade SM16:0 over other SM species (Fig. 3H) and the accumulation of SM16:0 in lysosomes upon pharmacological inhibition of ASM in wt neurons (Fig. 3I).

### Altered gene expression of SM metabolic enzymes in the brain of ASMko mice

**Fig. 4:**
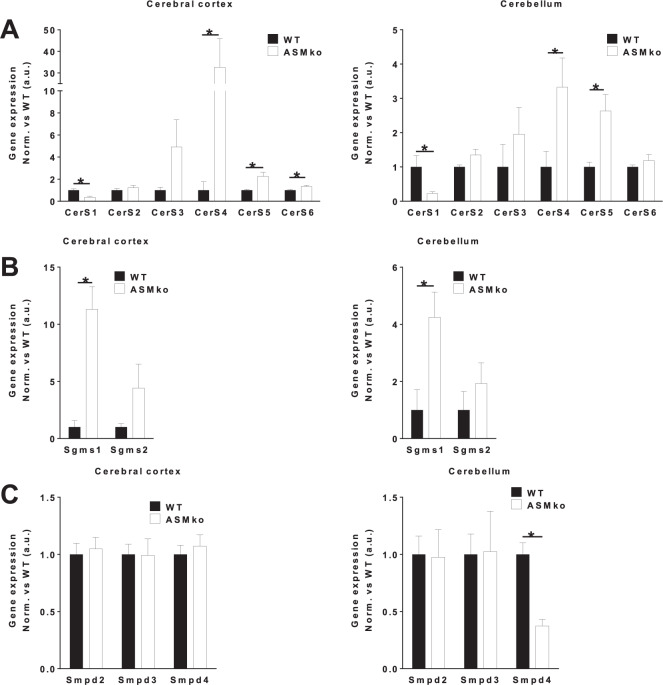
Altered gene expression of SM metabolic enzymes in the brain of ASMko mice. **A** Graphs show mean ± SEM expression levels of *CerS1-6* genes encoding for CerS1-6 measured by qPCR in cortical or cerebellar extracts of wt and ASMko mice. Data are expressed in arbitrary units normalized to the values obtained in wt mice that were considered 1 (*n* = 5; *p < 0.05). **B** Graphs show mean ± SEM expression levels of the *Sgms1-2* genes encoding for SMS1-2, measured by qPCR in cortical and cerebellar extracts of wt and ASMko mice. Data are expressed in arbitrary units normalized to the values obtained in wt mice that were considered 1 (*n* = 5; *p < 0.05). **C** Graphs show mean ± SEM expression levels of the *Smpd2-4* genes encoding for Smases2-4, measured by qPCR in cortical and cerebellar extracts of wt and ASMko mice. Data are expressed in arbitrary units normalized to the values obtained in wt mice that were considered 1. (*n* = 5; *p < 0.05).

The changes in the relative amounts of different SM species observed in the ASMko mice led us to analyze the gene expression of SM anabolic and catabolic enzymes. Quantitative PCR (qPCR) of the genes encoding for CerS1-6, SMS1-2 and Smases2-4 was performed in cortical and cerebellar extracts of wt and ASMko mice at 4.5 months of age (Fig. 4). In the ASMko cortex, CerS1 gene expression was 2.8-fold reduced while CerS4, 5 and 6 increased by 32.6, 2.2 and 1.3-fold, respectively; CerS2 and CerS3 did not change significantly compared to wt (Fig. 4A). In the ASMko cerebellum we observed similar reduction for CerS1 (4.5-fold) and increase for CerS4 (3.3-fold) and CerS5 (2.6-fold), while the levels of CerS 2, 3 and 6 did not change (Fig. 4A). Surprisingly, the expression of the genes encoding for SMS (Sgms1-2) was not decreased despite the accumulation of SMs. In contrast, an increase of Sgms1 was observed in both cortical (11.3-fold) and cerebellar (4.2-fold) extracts of ASMko mice compared to wt (Fig. 4B). We did not find significant changes in the expression of the genes encoding for Smases, except for the 2.7-fold reduction of *SMPD4* in the cerebellum (Fig. 4C).

### Genetic silencing of CerS5 prevents lysosomal damage and oxidative stress in cultured ASMko neurons

**Fig. 5:**
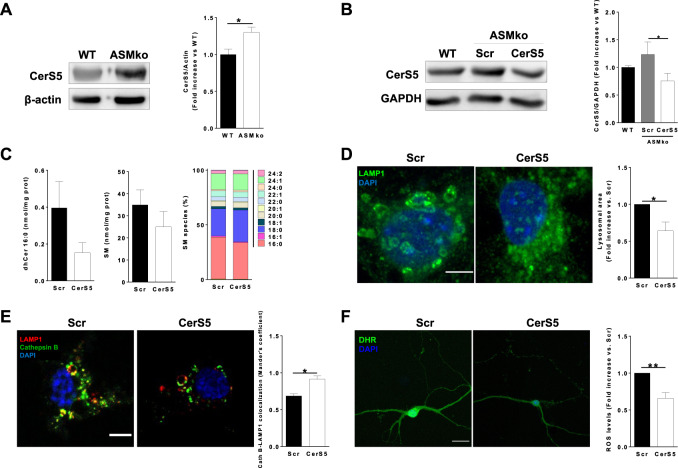
Beneficial effects of CerS5 genetic silencing in ASMko cultured neurons. **A** Western blot images of the levels of CerS5 and β-actin in cerebellar extracts of wt and ASMko mice. Graph shows mean ± SEM CerS5 protein expression in the ASMko cerebellar extracts normalized to β-actin and expressed as fold-increase with respect to wt extracts (*n* = 3; *p < 0.05). **B** Western blot images of the levels of CerS5 and GAPDH in non-infected wt cultured neurons or in ASMko cultured neurons infected with adenovirus containing either shRNA-CerS5 or shRNA-scramble. Graph shows mean ± SEM CerS5 protein expression normalized to GAPDH and expressed as fold-increase with respect to wt cultures (*n* = 3; *p < 0.05). **C** Graphs show mean ± SEM levels of dihydroCeramide 16:0 (left panel) and total SM (middle panel) expressed as nmol/mg protein or mean percentages of SM species with respect to total SM (right panel) in cultured ASMko neurons infected with adenovirus containing either shRNA-CerS5 or shRNA scramble (*n* = 4 independent cultures). **D** Immunocytochemical analysis against the lysosomal marker LAMP1 in cultured ASMko neurons infected with adenovirus containing either shRNA-CerS5 or shRNA-scramble. Graph shows mean ± SEM lysosomal area expressed as fold-change with respect to the shRNA-scramble infected cultures (*n* = 4 independent cultures; *p < 0.05). DAPI staining shows cell nuclei. Bar = 5 µm. **E** Immunocytochemical analysis against the lysosomal resident protein Cathepsin B and the lysosomal marker LAMP1 in cultured ASMko neurons infected with adenovirus containing either shRNA-CerS5 or shRNA-scramble. Graph shows mean ± SEM Mander’s colocalization coefficient between Cathepsin B and LAMP1 (*n* = 3 independent cultures; *p < 0.05). DAPI staining shows cell nuclei. Bar = 5 µm. **F** Representative images of DHR staining in cultured ASMko neurons infected with adenovirus containing either shRNA-CerS5 or shRNA-scramble. Graph shows mean ± SEM DHR intensity, proportional to ROS levels, expressed as fold-change with respect to shRNA-scramble infected cultures (*n* = 4 independent cultures; **p < 0.01). DAPI staining shows cell nuclei. Bar = 20 µm.

Among the changes in SM metabolic enzymes observed in the ASMko brains was the increase in CerS5, which is involved in the production of SM16:0 [4]. We confirmed by Western blot that, in agreement with its enhanced gene expression (Fig. 4A), the protein levels of CerS5 were 1.3-fold increased in the cerebellum of ASMko mice compared to wt (Fig. 5A). The increased CerS5 gene and protein expression together with the high levels and toxicity of SM16:0 found in the ASMko brain and neurons led us propose CerS5 inhibition would have beneficial effects. Since compounds that specifically inhibit this enzyme are not currently available, we assessed its genetic inhibition by RNA silencing. To this aim we infected ASMko cultured cortical neurons with adenovirus containing either shRNA-CerS5 or shRNA-scramble as a control. The efficacy of the treatment was confirmed by the 1.6-fold reduction in the CerS5 levels found by Western Blot in the shRNA-CerS5 compared to the shRNA-scramble infected ASMko cultures (Fig. 5B). This strategy rendered CerS5 amount in ASMko neurons to levels similar to those found in wt neurons (Fig. 5B). We also observed a 2-fold decrease in the levels of dihydroceramide16:0, which is a direct metabolite of CerS5 (Fig. 5C). In support of the targeting of the CerS5 silencing on SM16:0, this approach reduced by 1.2-fold the levels of this SM species in ASMko neurons and raised SM18:0 by 1.2-fold. It had no effect on SM24:1 (Fig. 5C). Total levels of SM were also not significantly changed by CerS5 silencing (Fig. 5C). Lysosomal parameters were analyzed to determine the effect of the treatment in these organelles. CerS5 silencing reduced lysosomal size (1.6-fold) (Fig. 5D) and lysosomal permeabilization measured by co-localization of CathepsinB-LAMP1 (1.3-fold) (Fig. 5E) in ASMko cultured neurons. The treatment also diminished oxidative stress (1.5-fold reduction in DHR intensity) (Fig. 5F).

### Genetic silencing of CerS5 ameliorates pathological hallmarks in the brain of ASMko mice

**Fig. 6:**
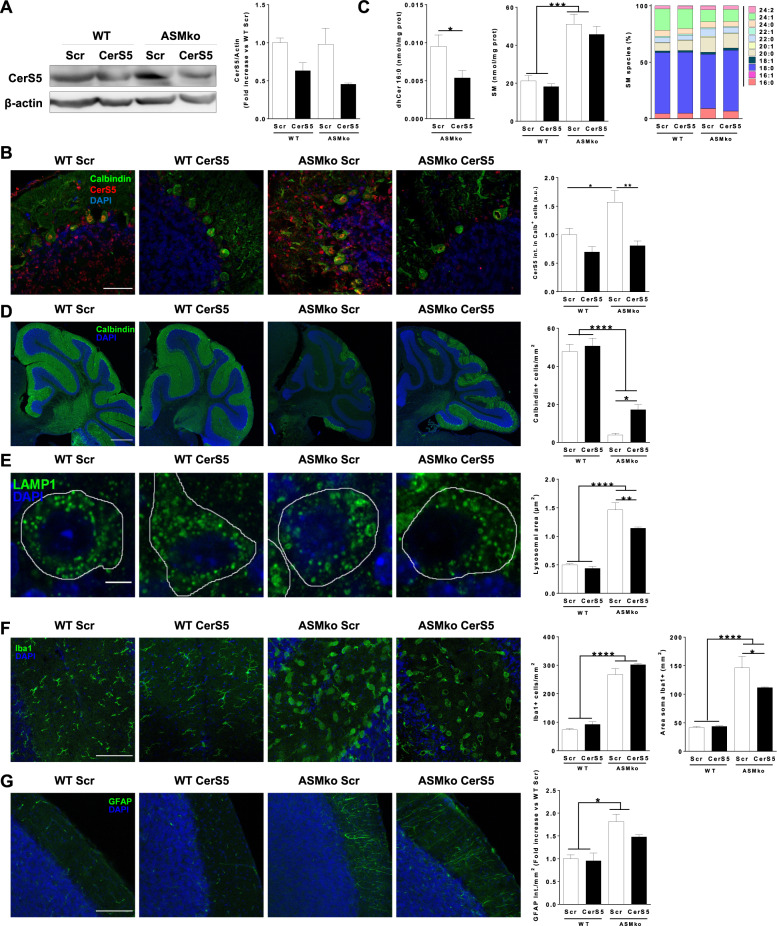
Beneficial effects of CerS5 genetic silencing in the cerebellum of ASMko mice. **A** Western blot images of the levels of CerS5 and β-actin in cerebellar extracts of wt and ASMko mice injected with AAV9-shRNA-CerS5 or AAV9-shRNA scramble. Graph shows mean ± SEM CerS5 protein expression in the cerebellar extracts normalized to β-actin and expressed as fold-change with respect to the shRNA scramble injected samples (*n* = 4). **B** Immunohistochemical analysis against the Purkinje cell marker Calbindin and CerS5 in the cerebellum of wt and ASMko mice injected with AAV9-shRNA-CerS5 or AAV9-shRNA-scramble. Graph shows mean ± SEM CerS5 intensity in Calbindin^+^ cells (*n* = 4; *p < 0.05; **p < 0.01). DAPI staining shows cell nuclei. Bar = 500 µm. **C** Graphs show mean ± SEM levels of dihydroCeramide 16:0 (left panel) and of total SM (middle panel) expressed as nmol/mg protein or mean percentages of SM species with respect to total SM (right panel) in cerebellar extracts of wt and ASMko mice injected with AAV9-shRNA-CerS5 or AAV9-shRNA-scramble (*n* = 4; *p < 0.05; ***p < 0.001). **D** Immunohistochemical analysis against the Purkinje cell marker Calbindin in the cerebellum of wt and ASMko mice injected with AAV9-shRNA-CerS5 or AAV9-shRNA-scramble. Graph shows mean ± SEM number of Purkinje cells (*n* = 4; *p < 0.05; ****p < 0.0001). DAPI staining shows cell nuclei. Bar = 500 µm. **E** Immunohistochemical analysis against the lysosomal marker LAMP1 in Purkinje cells of the cerebellum of wt and ASMko mice injected with AAV9-shRNA-CerS5 or AAV9-shRNA-scramble. Graph shows mean ± SEM lysosomal area (*n* = 4; **p < 0.01; ****p < 0.0001). DAPI staining shows cell nuclei. Bar = 5 µm. **F** Immunohistochemical analysis against the microglia marker Iba1 in the cerebellum of wt and ASMko mice injected with AAV9-shRNA-CerS5 or AAV9-shRNA-scramble. Graphs show mean ± SEM microglia number or area (*n* = 4; *p < 0.05; ****p < 0.0001). DAPI staining shows cell nuclei. Bar = 100 µm. **G** Immunohistochemical analysis against the astrocytic marker GFAP in the cerebellum of wt and ASMko mice injected with AAV9-shRNA-CerS5 or AAV9-shRNA-scramble. Graphs show mean ± SEM intensity associated to GFAP per area unit (*n* = 4; *p < 0.05). DAPI staining shows cell nuclei. Bar = 100 µm.

The positive effects of CerS5 genetic silencing in cultured ASMko neurons encouraged us to assess this strategy in the ASMko mice. To this aim, we used adeno associated serotype 9 viral vectors (AAV9) containing shRNA-scramble or shRNA-CerS5. We injected 5 × 10^12^ VG/ml AAV9- shRNA-scramble or AAV9-shRNA-CerS5 in the earliest and most affected brain area in the disease, the cerebellum, of wt and ASMko mice at 2 months of age. Seven weeks later different cellular and molecular analyses were carried out in the cerebellum. The silencing efficacy was confirmed by Western blot of the levels of CerS5 which were 1.6-fold and 2.2-fold reduced in wt and ASMko mice infected with AAV9-shRNA-CerS5 compared to the AAV9- shRNA-scramble infected (Fig. 6A). By immunostaining we determined that the increase in CerS5 protein levels in the ASMko mice compared to wt, which was counteracted by the AAV9-shRNA-CerS5 treatment (Fig. 6B), was cell-type specific concerning cerebellar neurons but not microglia or astrocytes (Supplementary Fig. 2A). AAV9-shRNA-CerS5 treatment did not modify the levels of other ceramide synthases such as CerS6, which expression was not found altered in ASMko mice compared to wt (Supplementary Fig. 2B). LC/MS analysis in cerebellar extracts showed that CerS5 silencing in the ASMko mice reduced by 1.8-fold the levels of dihydroCer16:0 and, while not changing significantly total SM levels, it prevented by 1.4-fold the SM16:0 increase (Fig. 6C). Thus, the amount of SM16:0 represented 4% of total SM in the cerebellum of wt mice, raised to 9% in the AAV9-shRNA-scramble injected ASMko mice and was reduced to 6% in the ASMko mice injected with AAV9-shRNA-CerS5 (Fig. 6C). In the AAV9-shRNA-CerS5 injected ASMko mice, the density of Purkinje cells, a type of neuron especially vulnerable in the disease, increased by 4.5-fold compared to the AAV9- shRNA-scramble injected ASMko mice as determined by immunohistochemical analysis with the specific marker Calbindin (Fig. 6D). The lysosomal size was reduced by 1.3-fold in the Purkinje cells as assessed by immunohistochemical analysis against LAMP1 (Fig. 6E). In addition, we observed the reduction of microglia size, detected with the marker iba1 (1.3-fold) (Fig. 6F), and of astrocyte reactivity, detected with the marker GFAP (1.2-fold) (Fig. 6G) in the AAV9-shRNA-CerS5 injected compared to the AAV9- shRNA-scramble injected ASMko mice.

### High levels of SM16:0 in plasma correlate with brain pathology in ASMko mice

**Fig. 7:**
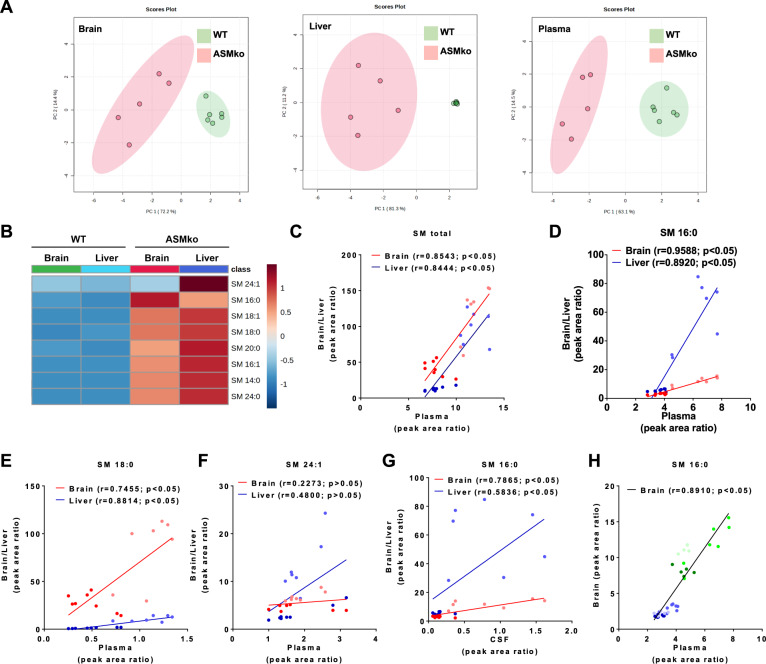
Increased SM 16:0 levels in plasma correlate with brain pathology in ASMko mice. **A** Multivariant analysis (Principal Component Analysis) of the SM species levels in brain, liver and plasma of wt and ASMko mice after autoscaling (mean centered and divided by the standard deviation of each SM species). Each symbol corresponds to an individual mouse (*n* = 5–6). **B** Heatmap showing the relative increments in the SM species levels in liver and brain of ASMko compared to wt mice after autoscaling (mean centered and divided by the standard deviation of each SM species) (*n* = 5–6). **C**–**F** Scatter plots together with regression lines showing the levels of total SM (**C**), SM16:0 (**D**), SM 18:0 (**E**) and SM24:1 (**F**) in plasma (*x*-axis) and brain or liver (*y*-axis) of wt and ASMko mice at 4.5 months of age. The correlation coefficient (r) and the *p* value of each correlation are indicated. Circles correspond to the values obtained in each individual mouse following the color code: dark blue (wt mice values in brain), light blue (ASMko mice values in brain), dark red (ASMko values in liver), light red (ASMko values in liver) (*n* = 7–8). *p < 0.05. **G** Scatter plots together with regression lines showing SM16:0 levels in CSF (*x*-axis) and brain or liver (*y*-axis) of wt and ASMko mice at 4.5 months of age. The correlation coefficient (r) and the *p* value of each correlation are indicated. Circles correspond to the values obtained in each individual mouse following the color code: dark blue (wt mice values in brain), light blue (ASMko mice values in brain), dark red (ASMko values in liver), light red (ASMko values in liver) (*n* = 7–8). *p < 0.05. **H** Scatter plots together with regression lines showing SM16:0 levels in plasma (*x*-axis) and brain or liver (*y*-axis) of wt and ASMko mice at 2, 4.5 and 6 months of age. The correlation coefficient (r) and the *p* value of each correlation are indicated. Circles correspond to the values obtained in each individual mouse following the color code: dark blue (wt 2 months old), medium blue (wt 4.5 months old), light blue (wt 6 months old), dark green (ASMko 2 months old), medium green (ASMko 4.5 months old), light green (ASMko 6 months old) (*n* = 5–8). *p < 0.05.

Currently, there is no biomarker specific for brain pathology in ASMD. Given the increase in the ASMko brains of SM16:0 compared to other SM species and its high toxicity for neurons, we postulated that this lipid might be a suitable indicator for neuronal damage in ASMD. To assess this possibility, we quantified by LC/MS the levels of SM species in brain, liver and blood samples taken from wt and ASMko mice at 4.5 months of age. The LC/MS data were analyzed by the MetaboAnalyst platform [28]. Further multivariate analysis (Principal Component Analysis) distinguished wt and ASMko mice based on the SM species levels in the three kinds of samples (Fig. 7A). Relative increments in the SM species levels in the liver and brain of ASMko compared to wt values were translated to a color code in a heatmap (Fig. 7B). This analysis unveiled clear differences between liver and brain in the ASMko animals. The increase in all SM species analyzed was higher in liver than in brain except for SM16:0, whose increment was higher in the brain than in the liver (Fig. 7B). In contrast, while SM24:1 showed the highest increment in the liver, its levels were unchanged in the ASMko brain compared to the wt situation (Fig. 7B). The LC/MS data on the levels of total SM and of SM species in brain and liver were correlated with those in plasma. The correlation coefficient (r) for plasma total SM was similar in brain (*r* = 0.8543) and liver (*r* = 0.8444), ruling out that this measurement is specific for brain pathology (Fig. 7C). In contrast, SM16:0 levels in plasma showed the highest correlation with the levels in brain (*r* = 0.9588) compared to liver (*r* = 0.8920) (Fig. 7D), while SM18:0 and SM24:1 plasma levels correlated better with liver (*r* = 0.8814 and *r* = 0.4800, respectively) than with brain values (*r* = 0.7455 and *r* = 0.2273, respectively) (Fig. 7E, F). Altogether, these results indicated that SM16:0 in plasma is the SM species that best reflects the changes in the ASMko brain. Levels of SM16:0 in the CSF also correlated better with those in brain (*r* = 0.7865) than in liver (*r* = 0.5836) although with higher variability among samples (Fig. 7G). In a recent cross-sectional study in ASMD patients, elevated levels in plasma of the de-acylated form of SM, lyso-sphingomyelin (LysoSM), were found positively associated with clinical severity [31]. We confirmed the remarkable LysoSM increase in the plasma of ASMko mice compared to wt that showed a similar correlation coefficient with both brain and liver levels (*r* = 0.8939 and *r* = 0.8948, respectively) (Supplementary Fig. 3A, B). As with total SM levels, this similar correlation does not support the utility of LysoSM to discriminate for brain pathology. Moreover, in contrast to SM 16:0, we did not find deleterious effects upon addition of LysoSM to cultured neurons (Supplementary Fig. 3C, D). Finally, to determine whether high SM16:0 plasma levels could also serve to monitor brain disease progression, brain and plasma samples from wt and ASMko mice at 2 months of age (when first motor symptoms appear) and at 6 months of age (end-stage disease) were analyzed by LC/MS and compared to those of 4.5 months of age. The results indicate that SM16:0 levels in plasma already present a high correlation with those in brain at early stages of the disease that is maintained until final stages (Fig. 7H).

## Discussion

Intracellular accumulation of different lipids is a hallmark in many lysosomal storage disorders such as ASMD [32]. Finding the primary offending lipid metabolite and its specific contribution to the disease are key questions in the field [33]. Answering these questions is relevant in the search for therapies that target pivotal steps in the pathogenic cascade and are as specific as possible minimizing side effects.

The aberrant accumulation of SM is a main pathological hallmark in ASMD responsible for many deleterious consequences in neurons [19]. However, differences in the fatty acid length and unsaturation degree lead to the existence of numerous SM species with particular distribution, metabolism and properties. We hypothesized that SM species also differ in their contribution to ASMD pathology and could individually become specific therapeutic targets. Our study has focused on the brain and neurons since the ERT strategy, which successfully treats the ASMD peripheral disease, does not address the neurological condition. The results here presented, using the ASMko mouse model for ASMD, point to SM16:0 as the SM species showing the highest relative accumulation and toxicity in ASMko neurons. In agreement with a relevant pathological role of SM16:0 its down regulation by genetic silencing of its metabolic enzyme CerS5 prevented aberrant phenotypes in vitro and in vivo.

The lipidomic analysis in the brain of wt and ASMko mice indicating the non-homogenous increase of SM species provided the first support to our hypothesis. The dramatic relative increase of SM16:0 compared to others species caught our attention. The availability of a fluorescently-traceable analogue to follow SM16:0 allowed us determining its lysosomal targeting in neurons. We propose that it is precisely this targeting what contributes to the high accumulation and toxicity of SM16:0 in the ASMD context. The gene mutated in the disease encodes for the lysosomal enzyme ASM. Indeed, we confirmed the higher efficacy of ASM to degrade SM16:0 over other SM species and the accumulation of SM16:0 in lysosomes upon pharmacological inhibition of ASM in wt neurons. Altogether these results support that ASM deficiency enhances SM16:0 accumulation in lysosomes promoting permeabilization and exocytosis of these organelles. In contrast, other SM species most abundant in the physiological situation such as the SM 24:1 would likely target other cellular compartments (i.e., plasma membrane). In these locations the neutral sphingomyelinase (NSmase), which gene is not affected in the disease, could degrade them preventing accumulation. In support of this view, NSmase have shown specificity for SM 24:1 [34] a SM species that showed no deleterious effects in the experiments we performed in neuronal cultures. Future work is required to answer questions such as why and how the different SM species target distinct subcellular compartments and which are the effects of the imbalance in their relative abundance.

The complex SM metabolism that involves numerous catabolic and anabolic enzymes provided the opportunity for specifically addressing SM16:0 accumulation and toxicity in ASMD. Six different CerS exist in mammalian cells that are responsible for the fatty acid composition of ceramides and therefore SMs [4]. Among them CerS5 is involved in the production of SM16:0 and is pretty enriched in brain tissue. Notably, CerS5 genetic silencing ameliorated ASMD phenotypes in ASMko cultured neurons and mouse brain without promoting a significant reduction of total SM levels. These results validate SM16:0 and CerS5 as specific therapeutic targets for brain pathology in ASMD.

Unexpectedly in the context of a disease characterized by increased SM levels, our results show elevated levels of enzymes that contribute to SM synthesis (i.e., CerS5 and SMS1) and no major changes in the catabolic enzymes. It might be that accumulation of SM in lysosomes, where the lipid should be degraded, triggers signals for SM synthesis up regulation despite the failure to eliminate it due to lysosomal impairment. The increments we observed in the mRNA levels of these enzymes point to the up regulation of their transcription as means to increase the protein levels. Whether and how the changes on the levels of different SM species affect transcription factors involved in the expression of their metabolic enzymes is another important question to address.

Finding biomarkers for neuronal damage is a challenge for neurological lysosomal storage disorders in which peripheral disease also takes place. A guideline for ASMD diagnosis was published taking into account the different subtypes of the disease that besides the infantile neurovisceral type A form also includes the chronic neurovisceral type A/B and the chronic visceral type B forms [35]. Hepatosplenomegaly and mixed dyslipidemia in blood are common features to all of them and have served to monitor the peripheral effects of the ERT in ASMD patients [21, 22]. A recent cross-sectional study in these patients indicated that plasma levels of the de-acylated form of SM, LysoSM, are positively associated with clinical severity [31]. However, biomarkers to follow neuronal damage are not available. Our results indicate that levels of SM16:0 in plasma show a very high correlation with those in brain while other SM species like SM18:0 correlate better with liver pathology. To explain how SM16:0 is released from brain into plasma we propose that the high lysosomal exocytosis induced by this lipid in neurons facilitates its cellular efflux and the incorporation into high-density lipoprotein (HDL) particles that can cross the BBB [36]. We cannot rule out that, in addition, BBB integrity is compromised when ASM is deficient.

In all, we believe the data here presented support the value of a particular SM species, SM16:0, both as a target for therapy and as early indicator for the fatal brain disease in ASMD.

## Supplementary information

checklist

Legends Supplementary Figures

Original Data File

Supplementary Figure 1

Supplementary Figure 2

Supplementary Figure 3

## Data Availability

The experimental data sets generated and/or analyzed during the current study are available from the corresponding author upon reasonable request. No applicable resources were generated during the current study.
