# Supplementary material for: Sphingomyelin 16:0 is a therapeutic target for neuronal death in acid sphingomyelinase deficiency
Source: Cell Death Dis. 2023 Apr 6;14(4):248. doi: 10.1038/s41419-023-05784-2 (PMC10079961; doi:10.1038/s41419-023-05784-2)
Supplement: Supplementary file 2 — Legends Supplementary Figures [file 41419_2023_5784_MOESM2_ESM.docx]

**Supplementary Figures**

**Supplementary Figure 1. SM16:0 toxic effects in cultured neurons are dose dependent and stronger in ASMko than in wt cells**

1. Graphs show mean ± SEM of total SM levels expressed as nmol/mg protein in cultured wt or ASMko neurons incubated with vehicle or with the indicated concentrations of SM16:0 (n=3 independent cultures; *p<0.05; **p<0.01)
2. DHR staining in cultured wt or ASMko neurons incubated with vehicle or with the indicated concentrations of SM16:0. DAPI staining shows cell nuclei. Graph shows mean ± SEM DHR intensity, proportional to ROS levels, expressed as fold-increase with respect to vehicle treated cultures (n=3 independent cultures; *p<0.05). Bar=20 µm
3. Graph shows mean ± SEM percentage of apoptotic cells measured by TUNEL assay in cultured wt or ASMko neurons incubated with vehicle or with the indicated concentration of SM16:0 (n=3 independent cultures; *p<0.05)

**Supplementary Figure 2. CerS5 does not localize in microglia or astrocytes and CerS6 levels in neurons are not affected by the silencing of CerS5**

1. Immunohistochemical analysis against markers of microglia or astrocytes, F4/80 and GFAP respectively, and CerS5 in the cerebellum of wt and ASMko mice injected with AAV9-shRNA-scramble. DAPI staining shows cell nuclei. Bar=100 µm.
2. Immunohistochemical analysis against the neuronal marker MAP2 and CerS6 in the cerebellum of wt and ASMko mice injected with AAV9-shRNA-CerS5 or AAV9-shRNA-scramble. Graph shows mean ± SEM CerS6 intensity in the neurons (n=4 independent mice of each group). DAPI staining shows cell nuclei. Bar=100 µm.

**Supplementary Figure 3. LysoSM accumulates to similar extent in brain, liver and plasma of ASMko mice and does not induce toxicity in cultured ASMko neurons**

1. Graph show mean ± SEM LysoSM levels in brain, liver and plasma from wt and ASMko mice. Data are expressed as percentage of wt samples (n=6; ***p<0.001; ****p<0.0001)
2. Scatter plots together with regression lines showing LysoSM levels in plasma (x-axis) and brain or liver (y-axis) of wt and ASMko mice at 4.5 months of age. The correlation coefficient (r) and the p value of each correlation are indicated. Circles correspond to the values obtained in each individual mouse following the colour code: dark blue (wt mice values in brain), light blue (ASMko mice values in brain), dark red (wt values in liver), light red (ASMko values in liver) (n=7-8; *p<0.05). Note dark blue and dark red circles lay close together at 0.00 value
3. Graphs show mean ± SEM of total SM levels in cultured wt neurons incubated with LysoSM expressed as percentage of vehicle treated cultures (n= 3 independent cultures)
4. Graph shows mean ± SEM cellular viability measured by MTT in cultured wt neurons incubated with vehicle or with LysoSM and expressed as percentage of living cells with respect to vehicle treated cultures (n= 3 independent cultures; p>0.05)
