# Supplementary figures and images for: Sphingomyelin 16:0 is a therapeutic target for neuronal death in acid sphingomyelinase deficiency
Source: Cell Death Dis. 2023 Apr 6;14(4):248. doi: 10.1038/s41419-023-05784-2 (PMC10079961; doi:10.1038/s41419-023-05784-2)

The labelled lanes are those cropped and shown in the indicated figures

**Fig 5A**

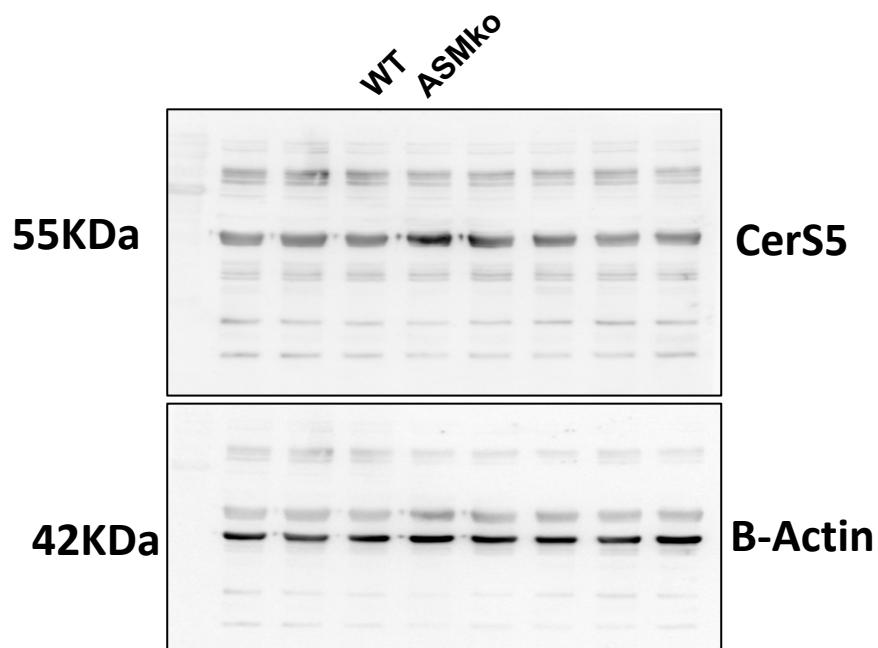

**Fig 5B**

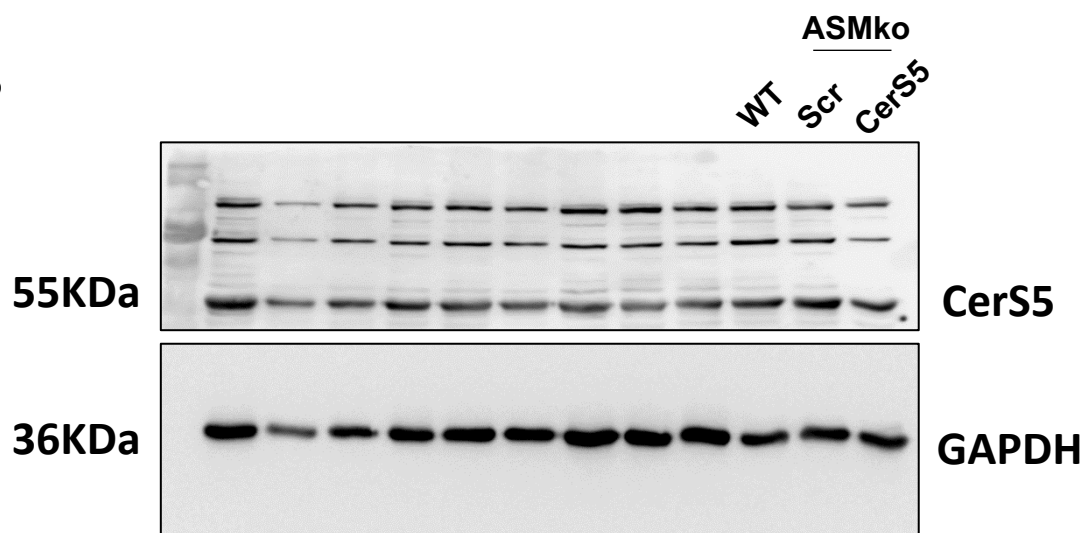

**Fig 6A**

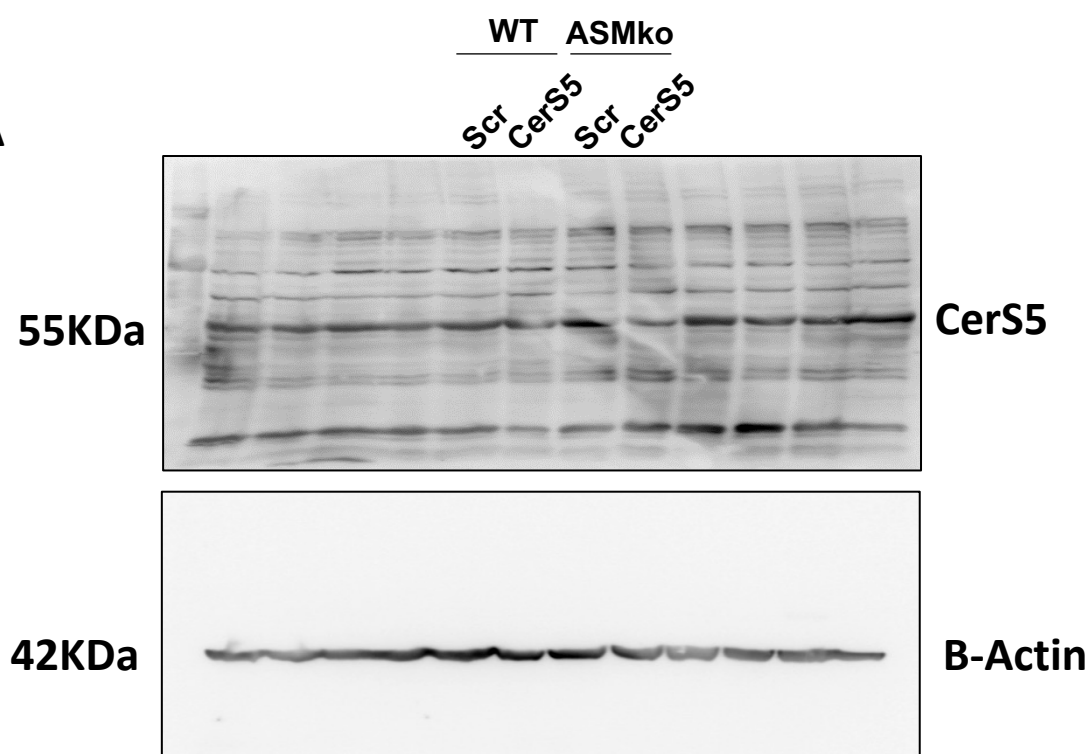

Supplement: Supplementary file 3 — Original Data File [file 41419_2023_5784_MOESM3_ESM.pdf]

# SUPPLEMENTARY FIGURE 1

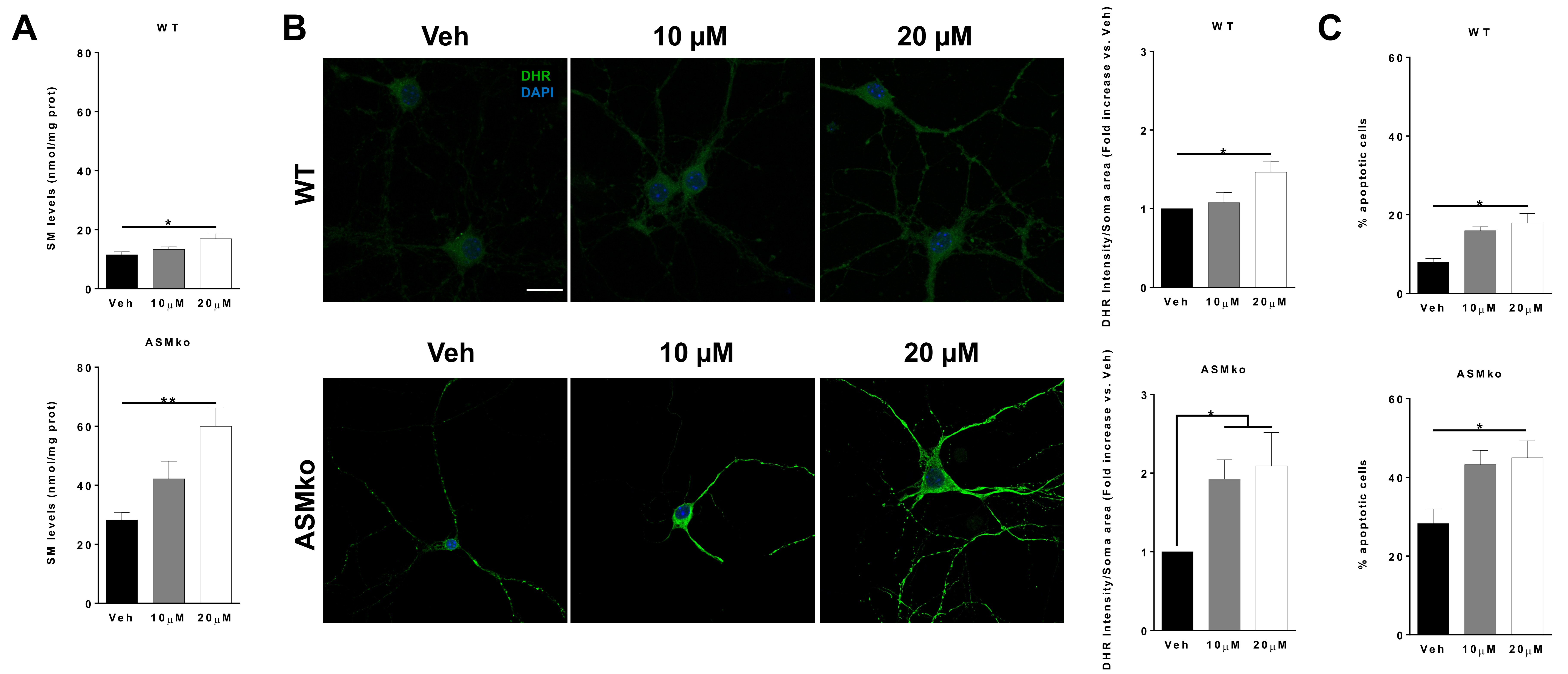

Supplement: Supplementary file 4 — Supplementary Figure 1 [file 41419_2023_5784_MOESM4_ESM.pdf]

SUPPLEMENTARY FIGURE 2

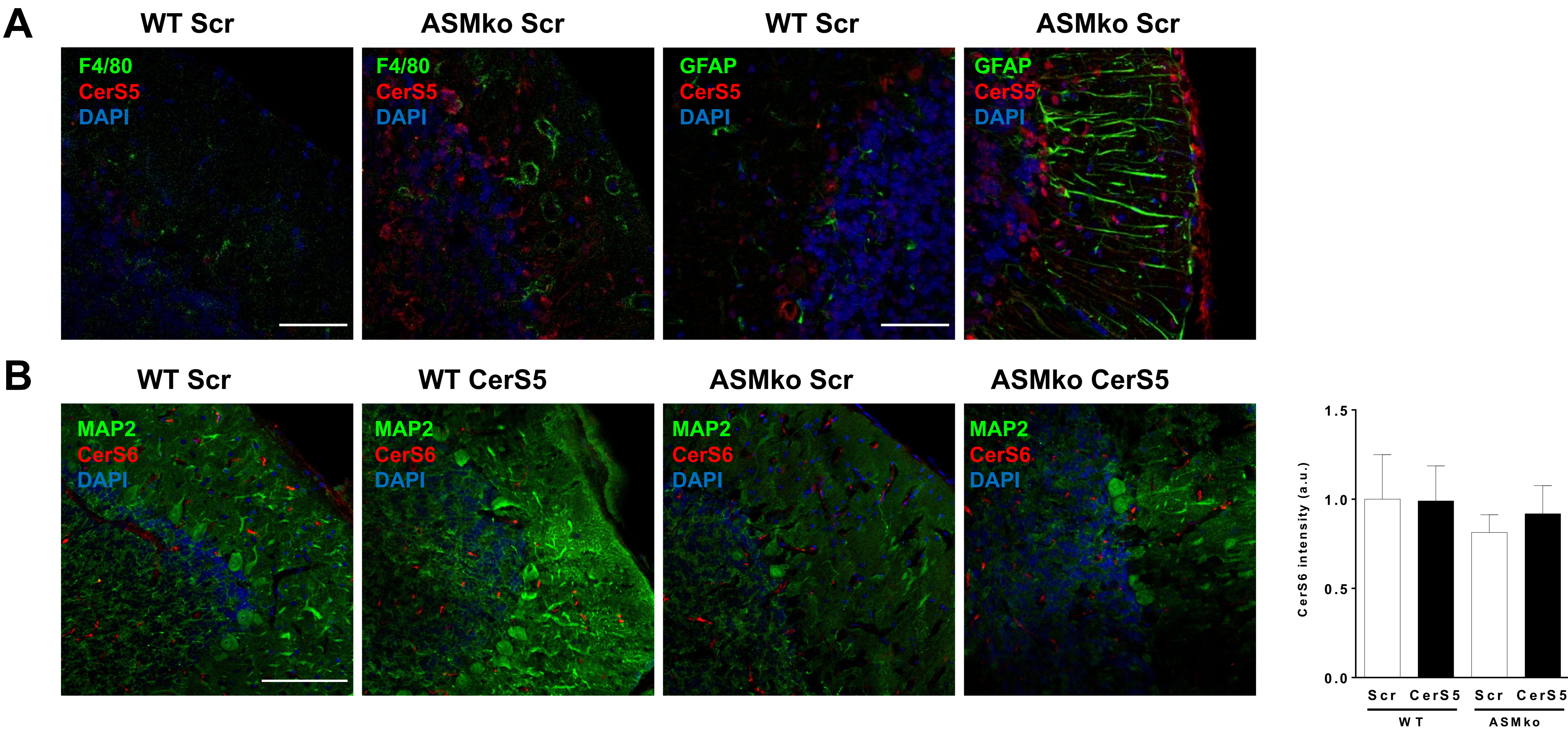

Supplement: Supplementary file 5 — Supplementary Figure 2 [file 41419_2023_5784_MOESM5_ESM.pdf]

SUPPLEMENTARY FIGURE 3

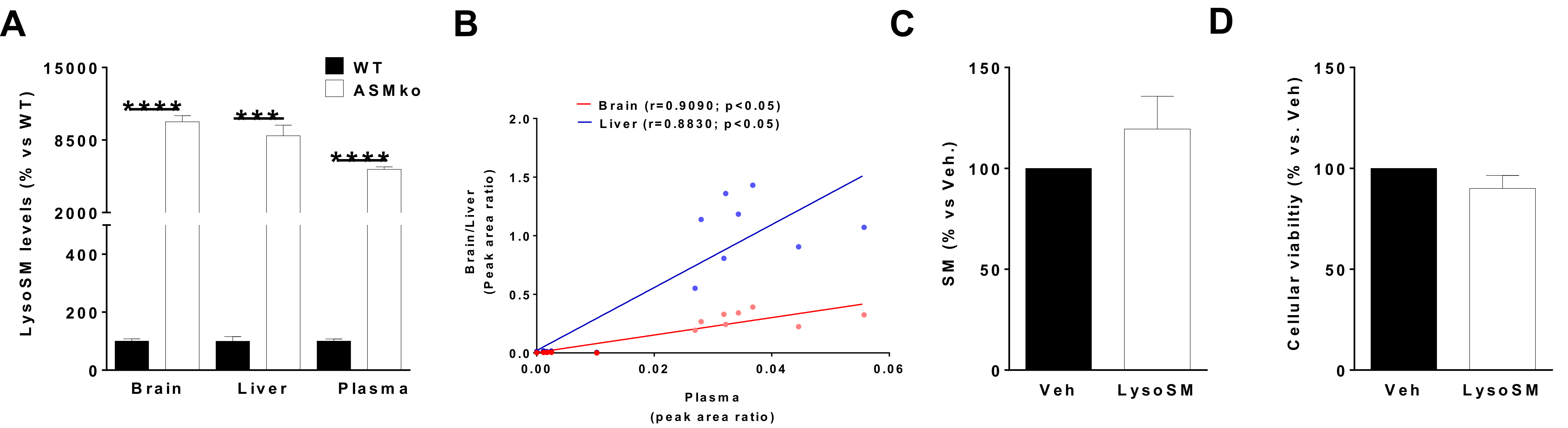

Supplement: Supplementary file 6 — Supplementary Figure 3 [file 41419_2023_5784_MOESM6_ESM.pdf]
